# Adoptive transfer of genetically engineered WT1-specific cytotoxic T lymphocytes does not induce renal injury

**DOI:** 10.1186/1756-8722-7-3

**Published:** 2014-01-06

**Authors:** Hiroaki Asai, Hiroshi Fujiwara, Sohei Kitazawa, Naoto Kobayashi, Toshiki Ochi, Yukihiro Miyazaki, Fumihiro Ochi, Yoshiki Akatsuka, Sachiko Okamoto, Junichi Mineno, Kiyotaka Kuzushima, Hiroaki Ikeda, Hiroshi Shiku, Masaki Yasukawa

**Affiliations:** 1 Department of Hematology, Clinical Immunology, and Infectious Diseases, Ehime University Graduate School of Medicine, Toon, Ehime, Japan; 2 Department of Molecular Pathology, Ehime University Graduate School of Medicine, Toon, Ehime, Japan; 3 Medical Education Center, Ehime University Graduate School of Medicine, Toon, Ehime, Japan; 4 Department of Hematology and Oncology, Fujita Health University, Toyoake, Aichi, Japan; 5 Center for Cell and Gene Therapy, Takara Bio Inc., Otsu, Shiga, Japan; 6 Division of Immunology, Aichi Cancer Center Research Institute, Nagoya, Aichi, Japan; 7 Department of Immuno-Gene Therapy, Mie University Graduate School of Medicine, Tsu, Mie, Japan

**Keywords:** Immunotherapy, WT1, Podocytes, Cytotoxic T lymphocytes

## Abstract

Because WT1 is expressed in leukemia cells, the development of cancer immunotherapy targeting WT1 has been an attractive translational research topic. However, concern of this therapy still remains, since WT1 is abundantly expressed in renal glomerular podocytes. In the present study, we clearly showed that WT1-specific cytotoxic T lymphocytes (CTLs) certainly exerted cytotoxicity against podocytes *in vitro*; however, they did not damage podocytes *in vivo*. This might be due to the anatomical localization of podocytes, being structurally separated from circulating CTLs in glomerular capillaries by an exceptionally thick basement membrane.

## Findings

Because WT1 is expressed in leukemia cells, including leukemia stem cells, the development of cell-mediated immunotherapy targeting WT1 has been an attractive translational research topic [1,2]. However, concern still remains about adverse events resulting from damage to normal tissues mediated by cytotoxic T lymphocytes (CTLs), since WT1 is also expressed in some lineages of normal cell as well as leukemia cells.

It is well known that WT1 is abundantly expressed in renal glomerular podocytes (or visceral epithelial cells) and that dysfunction of podocytes results in severe renal failure [3]. In addition, it has been recently reported that podocytes have functions of professional antigen-presenting cells [4]. Therefore, it seems important to clarify whether WT1-specific CTLs do not exert cytotoxicity against podocytes. In the present series of experiments, we examined in detail the cytotoxic effect of WT1-specific CTLs against podocytes using *in vitro* and *in vivo* systems.

## Methods

**Figure 1:**
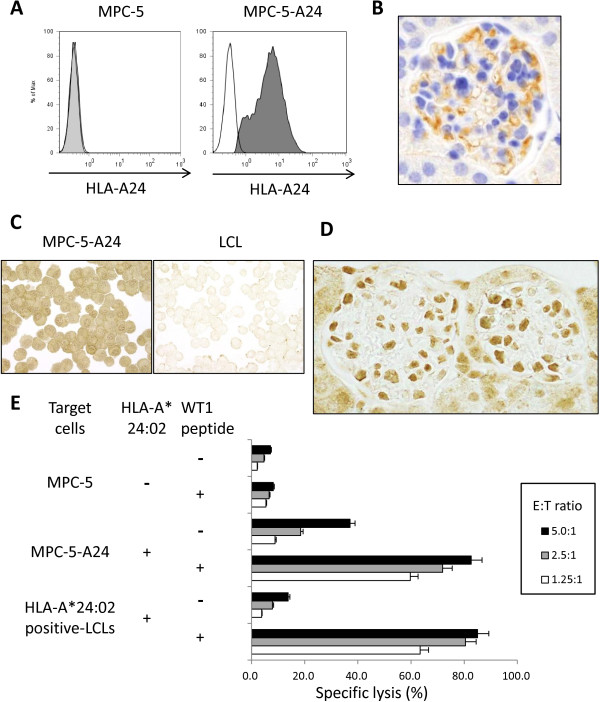
Cytotoxicity of WT1-specific and HLA-A*24:02-restricted CTLs against podocytes. (A) Expression of HLA-A24:02 on the HLA-A*24:02 gene-transduced mouse podocyte cell line, MPC-5 (MPC-5-A24). Flow cytometric analysis was performed using anti-HLA-A24:02 monoclonal antibody (One Lambda, Canoga Park, CA, USA). **(B)** Expression of HLA class I in the glomerulus of a HLA-A*24:02-transgenic mouse (original magnification ×400). Immunohistochemistry was performed using an anti-HLA class I framework monoclonal antibody (Abcam, Cambridge, UK). **(C)** Expression of WT1 in the HLA-A*24:02 gene-transduced mouse podocyte cell line, MPC-5-A24. MPC-5-A24 cells and LCL were stained with a rabbit anti-human and mouse WT1 polyclonal antibody (Santa Cruz Biotechnology, Dallas, TX, USA) (original magnification ×400). Notably, WT1 is abundantly expressed in MPC-5-A24 cells but not LCL. **(D)** Expression of WT1 in the glomerulus of a HLA-A*24:02-transgenic mouse (original magnification ×400). Immunohistochemistry was performed using a rabbit anti-human and mouse WT1 polyclonal antibody (Santa Cruz Biotechnology). **(E)** Cytotoxicity of WT1-specific and HLA-A*24:02-restricted CTLs against MPC-5, MPC-5-A24, and HLA-A*24:02-positive LCLs in the presence or absence of WT1 peptide at various effector:target cell ratios.

WT1-specific and HLA-A*24:02-restricted CTLs were generated by *T-cell receptor* (*TCR*) gene transfer using the novel retrovirus vector [5] into peripheral blood CD8^+^ T cells, as described previously [6]. We used a mouse podocyte cell line, MPC-5 [7], as the target cells, since there is a high homology between the human and mouse WT1 amino acid sequences, and WT1_235–243_ (CYTWNQMNL), which is the epitope of our WT1-specific CTLs, is completely conserved between the two species. The MPC-5 cells were transfected with the HLA-A*24:02 gene, as described previously with a slight modification [8]. As shown in Figure 1A, HLA-A*24:02 gene-transduced mouse podocytes expressed HLA-A24:02 molecules on their surface. We named this cell line MPC-5-A24.

HLA-A*24:02-transgenic mice were produced as reported previously [9]. All *in vivo* experiments were approved by the Ehime University animal care committee. As shown in Figure 1B, HLA-A24:02 molecules were expressed in the tissues of these transgenic mice, including glomeruli. HLA-A*24:02-transgenic mice were subsequently injected intravenously with 2.5 × 10^6^ WT1-specific and HLA-A*24:02-restricted CTLs or non-gene-modified CD8^+^ T cells (control CTLs). As we reported previously [6,10], the dose of *TCR* gene-engineered T cells used in the present study is enough to show anti-leukemia effect *in vivo*. Mice that had received WT1-specific CTLs and control CTLs were sacrificed after 7 days, and the presence of tissue damage was examined morphologically. Trafficking of WT1-specific CTLs in HLA-A*24:02-transgenic mice was examined using *luciferase* gene-transfected CTLs in a bioluminescence imaging assay as reported previously [10]. Serial acquisition of luciferase photon counts using luciferin was carried out on days 1, 3, and 6 using AEQUORIA (Hamamatsu Photonics, Hamamatsu, Japan), and analyzed using AQUACOSMOS software (Hamamatsu Photonics).

## Results

As shown in Figure 1C, WT1 appeared to be abundantly expressed in the HLA-A*24:02 gene-transduced mouse podocyte cell line, MPC-5-A24. We also confirmed that WT1 was abundantly expressed in podocytes of HLA-A*24:02-transgenic mice. (Figure 1D). Figure 1E shows the cytotoxicity of WT1-specific and HLA-A*24:02-restricted CTLs against various target cells. WT1-specific CTLs showed strong cytotoxicity against WT1_235–243_ peptide-loaded but not -unloaded HLA-A*24:02-positive LCLs. Notably, WT1-specific CTLs apparently exerted cytotoxicity against MPC-5-A24, and their cytotoxicity against WT1_235–243_ peptide-loaded MPC-5-A24 appeared to be higher than that against WT1 peptide-unloaded MPC-5-A24. In contrast, WT1-specific CTLs did not show cytotoxicity against WT1 peptide-loaded or -unloaded MPC-5. These results showed that WT1-specific CTLs can lyse podocytes in an HLA-restricted manner through recognition of the WT1 epitope that is naturally processed from WT1 protein in podocytes and presented on the cell surface in the context of HLA class I molecules.

**Figure 2:**
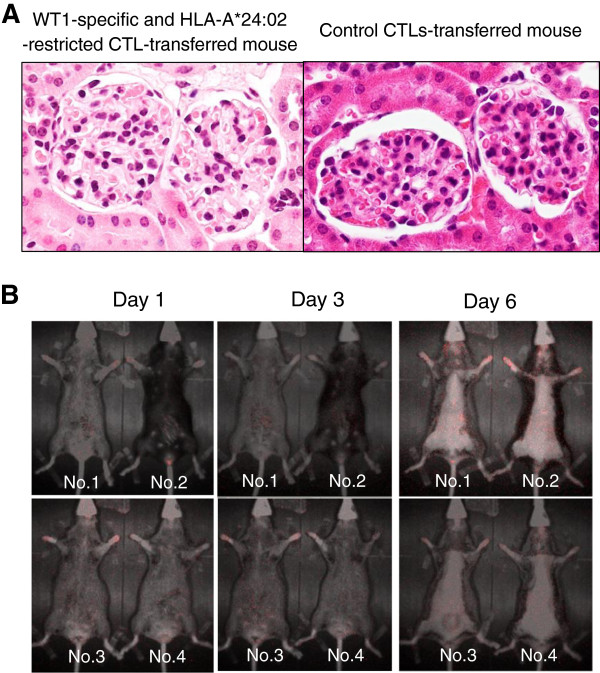
Monitoring of renal damage in HLA-A*24:02-transgenic mice following transfer of WT1-specific and HLA-A*24:02-restricted CTLs. (A) Histopathology of the glomeruli of HLA-A*24:02-transgenic mice that had received WT1-specific and HLA-A*24-restricted CTLs and control CTLs. (hematoxylin-eosin stain; original magnification x400). Notably, lymphocyte infiltration and tissue damage are not detectable in the glomerulus of the WT1-specific CTL-transferred mouse. **(B)** Trafficking of WT1-specific and HLA-A*24:02-restricted CTLs in HLA-A*24:02-transgenic mice. Four mice were transferred with WT1-specific and HLA-A*24:02-restricted CTLs. Notably, CTLs have not accumulated in specific organs, including the kidneys.

We monitored in detail the renal function of HLA-A*24:02-transgenic mice following transfer of WT1-specific CTLs. Body weight loss and severe proteinuria were not observed in mice that had received WT1-specific CTLs (data not shown). As shown in Figure 2A, lymphocyte infiltration or glomerular injury was not detectable morphologically in WT1-specific CTL-transferred mice. Also, damage of other organs, including pleura, was not detectable (data not shown). Finally, we examined the kinetic distribution of WT1-specific CTLs in HLA-A*24:02-transgenic mice. As shown in Figure 2B, WT1-specific and HLA-A*24:02-restricted CTLs did not accumulate in kidneys.

## Discussion

The present *in vitro* and *in vivo* studies clearly showed that WT1-specific CTLs indeed exerted cytotoxicity against renal glomerular podocytes in an HLA-restricted manner; *in vivo*, however, podocytes were able to escape from the cytotoxicity of WT1-specific CTLs. This might be due to the anatomical localization of podocytes, being located outside the capillaries of the glomerulus. Because podocytes are completely separated from capillaries in which CTLs are circulating by a thick glomerular basement membrane which can inhibit the pass of blood cells and even serum protein, CTLs cannot come into contact with podocytes under normal condition. However, in the patients with glomerulonephritis, the permeability of the glomerular basement membrane increases, resulting in proteinuria; therefore, CTLs may infiltrate through the basement membrane and damage podocytes. Therefore, in conclusion, adoptive transfer of WT1-specific CTLs in patients without renal failure is likely safe; however, it should be performed cautiously in patients with proteinuria.

## Abbreviations

CTLs: Cytotoxic T lymphocytes; TCR: T-cell receptor.

## Competing interests

The authors declare no competing interest.

## Authors’ contributions

HA, SK, TO, YM, and FO performed experiments and analyzed data. HF designed research and performed experiments. NK, SO, JM, KK, HI, and HS provided materials and discussed the experimental results. YA provided materials and performed experiments. MY designed research, wrote the manuscript, and provided financial support. All authors read and approved the final manuscript.
